# Neural activation changes following attention bias modification treatment or a selective serotonin reuptake inhibitor for social anxiety disorder

**DOI:** 10.1017/S0033291724001521

**Published:** 2024-09

**Authors:** Omer Azriel, Gal Arad, Niv Tik, Mark Weiser, Miki Bloch, Eddie Garber, Amit Lazarov, Daniel S. Pine, Ido Tavor, Yair Bar-Haim

**Affiliations:** 1 School of Psychological Sciences, Tel Aviv University, Tel Aviv, Israel; 2 Sackler Faculty of Medicine, Tel Aviv University, Tel Aviv, Israel; 3 Sagol School of Neuroscience, Tel Aviv University, Tel Aviv, Israel; 4 Department of Psychiatry, Sheba Medical Center, Tel Aviv, Israel; 5 Psychiatric Department, Tel Aviv Sourasky Medical Center, Tel Aviv, Israel; 6 Section on Developmental Affective Neuroscience, National Institute of Mental Health, Bethesda, MD, USA

**Keywords:** anterior cingulate cortex, attention bias modification, dorsolateral prefrontal cortex, inferior frontal gyrus, selective serotonin reuptake inhibitor, social anxiety

## Abstract

**Background:**

Delineation of changes in neural function associated with novel and established treatments for social anxiety disorder (SAD) can advance treatment development. We examined such changes following selective serotonin reuptake inhibitor (SSRI) and attention bias modification (ABM) variant – gaze-contingent music reward therapy (GC-MRT), a first-line and an emerging treatments for SAD.

**Methods:**

Eighty-one patients with SAD were allocated to 12-week treatments of either SSRI or GC-MRT, or waitlist (*n*s = 22, 29, and 30, respectively). Baseline and post-treatment functional magnetic resonance imaging (fMRI) data were collected during a social-threat processing task, in which attention was directed toward and away from threat/neutral faces.

**Results:**

Patients who received GC-MRT or SSRI showed greater clinical improvement relative to patients in waitlist. Compared to waitlist patients, treated patients showed greater activation increase in the right inferior frontal gyrus and anterior cingulate cortex when instructed to attend toward social threats and away from neutral stimuli. An additional anterior cingulate cortex cluster differentiated between the two active groups. Activation in this region increased in ABM and decreased in SSRI. In the ABM group, symptom change was positively correlated with neural activation change in the dorsolateral prefrontal cortex.

**Conclusions:**

Brain function measures show both shared and treatment-specific changes following ABM and SSRI treatments for SAD, highlighting the multiple pathways through which the two treatments might work. Treatment-specific neural responses suggest that patients with SAD who do not fully benefit from SSRI or ABM may potentially benefit from the alternative treatment, or from a combination of the two.

**Trial Registration:**

ClinicalTrials.gov, Identifier: NCT03346239. https://clinicaltrials.gov/ct2/show/NCT03346239

## Introduction

Social anxiety disorder (SAD), which involves fear of situations where one may be scrutinized (American Psychiatric Association, 2013), is common (Baxter, Patton, Scott, Degenhardt, & Whiteford, 2013; Kessler, Chiu, Demler, & Walters, 2005; Wittchen et al., 2011) and impairing (Aderka et al., 2012; Stein et al., 2017). Serotonin reuptake inhibitors (SSRIs) pharmacotherapy is an efficacious first-line treatment for SAD (Curtiss, Andrews, Davis, Smits, & Hofmann, 2017; Davis, Smits, & Hofmann, 2014; Mayo-Wilson et al., 2014), but many patients fail to respond to treatment (Blanco et al., 2010; Davidson et al., 2004).

Novel treatments that exploit technology and cognitive-neuroscience knowledge may help to address these challenges (Gober, Lazarov, & Bar-Haim, 2021; Mohr, Burns, Schueller, Clarke, & Klinkman, 2013). One such treatment is gaze-contingent music reward therapy (GC-MRT), an eye-tracking-based attention bias modification (ABM) protocol designed to target threat-related attention in SAD (Armstrong & Olatunji, 2012; Bar-Haim, Lamy, Pergamin, Bakermans-Kranenburg, & van IJzendoorn, 2007; Lazarov, Abend, & Bar-Haim, 2016; Lazarov, Pine, & Bar-Haim, 2017; Pergamin-Hight, Naim, Bakermans-Kranenburg, van IJzendoorn, & Bar-Haim, 2015). In GC-MRT, patients' gaze is monitored during free viewing of matrices consisting of disgusted and neutral faces. A music track selected by each patient plays during fixations on neutral faces but stops during fixation on disgusted faces. Thus, via gaze-contingent operant conditioning, patients gradually learn to allocate their attention to neutral over threat faces. Compared to previous ABM protocols, applying variations of the dot-probe task (MacLeod, Mathews, & Tata, 1986), GC-MRT's free-viewing matrix task holds better internal consistency and test–retest reliability, offering clearer view of underlying mechanisms and target engagement (Lazarov et al., 2016, 2017; Shamai-Leshem et al., 2023). Importantly, GC-MRT shows favorable clinical outcomes: two open trials of GC-MRT in adults (Umemoto et al., 2021) and children (Linetzky, Kahn, Lazarov, Pine, & Bar-Haim, 2019) with SAD reported a significant pre-to-post clinical improvement with large effect sizes (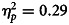 and Cohen's *d* = 1.43, respectively), high treatment adherence, and treatment credibility. One randomized controlled trial (RCT) (Lazarov et al., 2017) indicated greater efficacy of GC-MRT relative to a stringent control, and a second RCT (Arad et al., 2023) found comparably greater symptom reductions in GC-MRT and SSRI relative to a waitlist control, with no difference in efficacy between them. These RCTs also demonstrated cognitive target engagement (i.e. a reduction in dwell time on threatening stimuli), and a near transfer-of-learning effect in the presence of a new set of face stimuli not used in training. Here, we examine treatment-related changes in brain function during an implicit threat processing task in this second RCT before and after GC-MRT, SSRI, or a wait period.

Prior work suggests that SSRI treatment alters neural responses to social threats in the amygdala (Faria et al., 2012; Furmark et al., 2005, 2002; Giménez et al., 2014; Gingnell et al., 2016; Klumpp & Fitzgerald, 2018; Phan et al., 2013), insula (Giménez et al., 2014; Schneier, Pomplun, Sy, & Hirsch, 2011), anterior cingulate cortex (ACC) (Burkhouse et al., 2018; Giménez et al., 2014; Schneier et al., 2011), medial prefrontal cortex (mPFC) (Burkhouse et al., 2018; Giménez et al., 2014; Phan et al., 2013), hippocampus (Pantazatos, Talati, Schneier, & Hirsch, 2014), and temporal cortex (Pantazatos et al., 2014; Phan et al., 2013; Schneier et al., 2011). These studies used various threat processing paradigms, ranging from public speaking challenges to cognitive tasks applying visual presentations of socially threatening faces or words. Within these tasks, a distinction is made between tasks involving explicit emotion processing, where participants rate or respond to an emotional feature of a stimulus (e.g. indicating whether two faces express a similar emotion); and tasks involving implicit emotion processing, where stimuli contain emotional features, but participants do not rate or respond to these features. Importantly, these two types of tasks engage different neuro-cognitive processes (Critchley et al., 2000; Fusar-Poli et al., 2009; Mathersul et al., 2009; Norman, Polyn, Detre, & Haxby, 2006; Pantazatos, Talati, Pavlidis, & Hirsch, 2012). Whereas most of the above-mentioned studies applied explicit threat processing tasks (Burkhouse et al., 2018; Gingnell et al., 2016; Phan et al., 2013; Schneier et al., 2011), Pantazatos et al. (2014) used a task involving implicit processing of social threat. The neural findings associated with these two types of tasks may differ. For example, Pantazatos et al. (2014) reported functional changes in the hippocampus, not evident in studies applying explicit threat processing tasks to test SSRI's neural effects.

Data on ABM-associated changes in neural responses to social threats also indicate altered amygdala response (Britton et al., 2014; Månsson et al., 2013; Taylor et al., 2013), and changes in neural activation within the PFC (Browning, Holmes, Murphy, Goodwin, & Harmer, 2010; Eldar & Bar-Haim, 2010; Shechner & Bar-Haim, 2016; Taylor et al., 2013). Here too, the variability in neural findings may be partly attributed to differences in methods applied to manipulate threat processing. For example, whereas Månsson et al. (2013) and Taylor et al. (2013) used explicit matching of facial expressions, Hariri et al. (2002), Britton et al. (2014) and Eldar and Bar-Haim (2010) assessed neural responses during performance on the dot-probe task, involving implicit threat processing. Additionally, unlike the above-mentioned tasks, in the dot-probe task the presentation of faces is followed by a target, thus the faces and the target stimulus are not processed simultaneously. Browning et al. (2010) used a task in which implicit emotion processing took place. This task also involved direct (respond to faces) and indirect (respond to bars appearing alongside faces) processing. Importantly, this combination of implicit emotion processing during allocation of attention toward or away from faces makes it highly relevant for testing neural correlates of GC-MRT – an ABM protocol facilitating the modification of attention toward and away from faces using operant conditioning which is implicitly based on the faces' emotional valence.

Two studies specifically focused on neural correlates of GC-MRT (Umemoto et al., 2021; Zhu et al., 2023). These studies, however, examined pre-treatment predictors of clinical improvement. To our knowledge, no studies reported on pre- to post-GC-MRT treatment changes in neural activation during threat processing.

The current study examines common and specific effects of GC-MRT and SSRI pharmacotherapy on brain function during implicit social threat processing in patients with SAD. Functional magnetic resonance imaging (fMRI) data was collected at baseline and post-treatment, or an equivalent waiting period in a control group. To focus analyses on changes in neural activation when participants perform task demands that either match or contradict trained attentional patterns in GC-MRT, we used a task adapted from (Browning et al., 2010), and followed their analytic approach. Treatment-related changes in neural activation were tested by placing conflicting demands on attention. To this end, a computation of a training incongruent *v.* training congruent signal contrast was performed, reflecting brain response to conditions that are incompatible with the GC-MRT training direction. Compared to trials in which the direction of patients' attention conforms with their training, trials in which the attentional demands conflict with the training are generally expected to elicit greater activation in brain regions associated with attention control (Bishop, Duncan, Brett, & Lawrence, 2004; Browning et al., 2010; MacDonald, Cohen, Andrew Stenger, & Carter, 2000). The following hypotheses were tested in relation to this contrast:

Hypothesis 1: We expected that relative to the waitlist control, GC-MRT and SSRI would alter function within a neural circuit in which aberrant functioning was previously reported in SAD, and which has been suggested to be affected following SAD treatment (Brühl, Delsignore, Komossa, & Weidt, 2014; Etkin & Wager, 2007). Specifically, considering existing evidence on neural activation during implicit emotion processing in SAD (Blair et al., 2011, 2008; Gentili et al., 2008; Klumpp, Post, Angstadt, Fitzgerald, & Phan, 2013b), the effect of treatment on it (Britton et al., 2014; Burkhouse et al., 2018, 2017; Eldar & Bar-Haim, 2010; Pantazatos et al., 2014), and the relevance of the tested contrast to visual processing and attention control functions (Browning et al., 2010), we expected that these alterations would occur in parieto-occipital regions, ACC, and PFC. Evidence on the directionality of change is inconsistent, with some studies reporting increases, others reporting decreases, and some reporting no changes in activation within these regions. Given the diversity of brain regions involved, the inconsistent findings concerning change direction, and the limited previous evidence on GC-MRT-related neural changes, we decided to use a conservative whole brain analysis to identify treatment-related changes in the current study.

Hypothesis 2: GC-MRT and SSRI would induce distinct treatment-specific functional changes. Considering the reported neural correlates of ABM for SAD (Browning et al., 2010; Eldar & Bar-Haim, 2010; Taylor et al., 2013; Umemoto et al., 2021; Zhu et al., 2023), and the role of attention control in the mechanisms underlying ABM (Shechner & Bar-Haim, 2016), we expected that GC-MRT will be associated with alterations in the ACC and PFC, two key areas associated with attention control. In addition, based on Browning et al. (2010), we expected greater recruitment of these areas at post-treatment in GC-MRT patients when they are specifically required to direct attention opposite to their trained direction (i.e. the incongruent>congruent contrast), reflecting greater effort to control attention.

Hypothesis 3: Within the GC-MRT group, greater clinical improvement would relate to greater functional change in areas associated with attention control.

Additional separate exploratory analyses were conducted to examine changes in neural response to threat compared to neutral faces occurring during direct face processing (i.e. when patients are instructed to process faces) and indirect face processing (i.e. when patients are instructed to process bars appearing alongside faces).

## Methods and materials

### Participants

For the full protocol, sample-size determination, randomization, and blinding details see Arad et al. (2023) (ClinicalTrials.gov Identifier: NCT03346239). Of the 105 enrolled patients, 8 were ineligible for MRI procedures, 15 did not provide post-treatment MRI data, and one was excluded due to low accuracy on the task (<70%). Pre-to-post analyses (162 MRI scans) were conducted on 81 participants (mean [s.d.] age = 29.62 [7.05] years, 49 females): GC-MRT (*n* = 29), SSRI (*n* = 22), and waitlist control [WL, *n* = 30] (see online Supplementary Fig. S4). Groups did not differ in demographic or clinical characteristics at baseline (see online Supplementary Table S3). No patients in the GC-MRT group received SSRI treatment during their participation in the trial. Participants provided a written informed consent as approved by the local IRBs.

### Treatments

*SSRI treatment* followed a standard 12-week protocol of Escitalopram (Kasper, Stein, Loft, & Nil, 2005). This flexible dose protocol started from 5 mg and increased to 20 mg according to patients' response. Meetings with a psychiatrist occurred at weeks 1, 3, 6, and 12 of treatment.

For a detailed description of the *GC-MRT* protocol see Arad et al. (2023). Ten GC-MRT sessions were delivered – 8 twice-weekly over four weeks and two additional sessions at weeks 8 and 11. For each session patients selected a music track they wanted to listen to. Patients then viewed 30 matrices comprised of 16 faces each – 8 disgust (threat) faces and 8 neutral faces. Faces were taken from the Karolinska Directed Emotional Faces database (KDEF; Lundqvist, Flykt, and Öhman, 1998). Gaze was tracked throughout, and music was played only when fixating on neutral faces and stopped when fixating on disgusted faces. This gaze-contingent operant conditioning procedure induces attentional preference for neutral over threat faces.

The WL group received 8 sessions of GC-MRT, delayed by 12 weeks.

### Neuroimaging attention task

**Figure 1.**
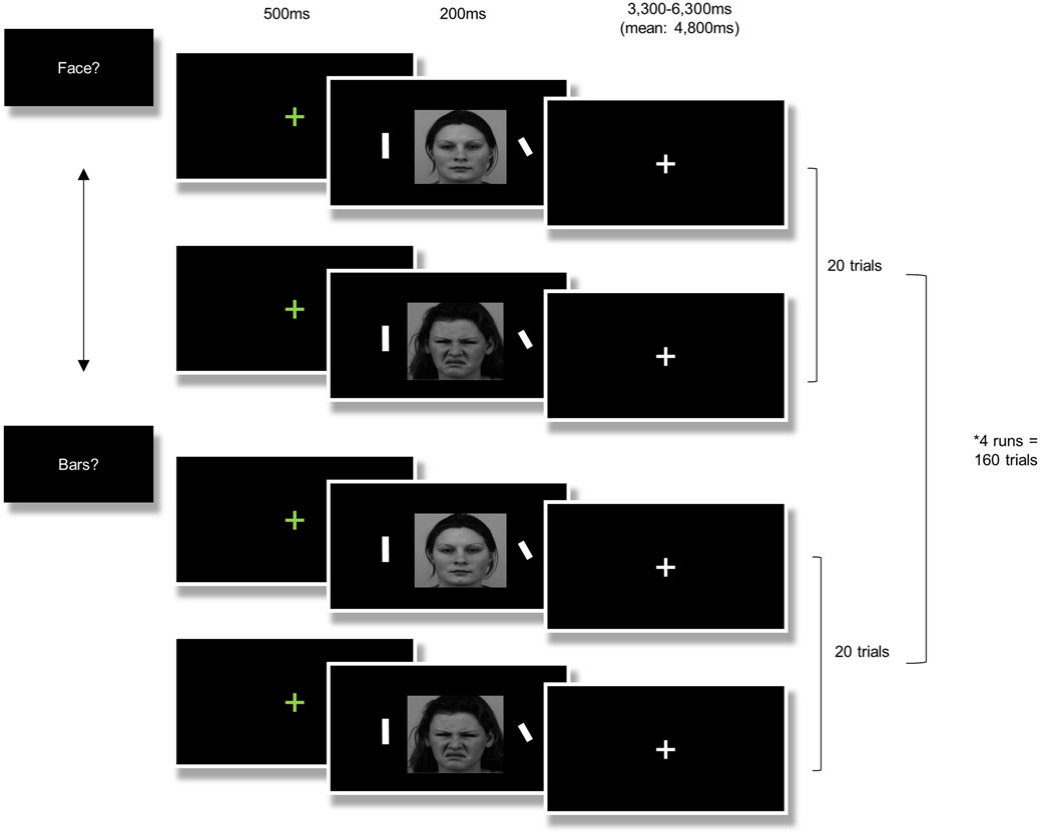
During the fMRI task, participants were instructed to indicate either the gender of the face or whether the flanking bars are aligned. In each block, half of the trials included a disgusted face and half of the trials included a neutral face. fMRI, functional magnetic resonance imaging.

A task adapted from Browning et al. (2010) was used (Fig. 1). Each trial began with a fixation cross (500 ms), followed by a face flanked by two bars (200 ms). Participants' attention was manipulated by instructing them to either identify the gender of the face (‘attend face’ blocks) or determine whether the flanking bars are aligned (‘attend bars’ blocks). Eight blocks of 20 trials each (160 total) were presented. In each block disgusted and neutral faces appeared with equal frequency. Faces were taken from the KDEF database (Lundqvist et al., 1998); importantly, actors that appeared in the attention task were different from actors that appeared in GC-MRT training. Task structure was factorial: two levels of emotion (disgust/neutral) by two levels of attention (toward/away from the presented face). This enabled an analysis contrasting brain function during conditions *congruent* with GC-MRT training (attending toward neutral faces and away from threat faces) with brain function during conditions *incongruent* with GC-MRT training (attending toward threat faces and away from neutral faces). Participants had up to 3250 ms to respond, followed by a jittered inter-trial interval ranging 50–3050 ms (mean duration 1550 ms). Every two blocks (‘attend face’/‘attend bars' presented in a random order) were performed within a single run with a short break between runs. The stimuli set used in the fMRI task differed from the set used during GC-MRT sessions to avoid a potential effect of familiarity on neural response. The task was run using Presentation (Neurobehavioral Systems Inc., USA, http://www.neurobs.com/). For a pre-treatment manipulation check and test-retest reliability of the task, see online Supplementary Material.

### Image acquisition and preprocessing

MRI data was acquired on a Siemens Magnetom Prisma 3 T scanner (Siemens, Erlangen, Germany), using a 64 channel phased array head coil. The protocol comprised structural and functional MRI scans as following: high resolution anatomical localizer image (MPRAGE): TR = 1750 ms, TE = 2.61 ms, TI = 900 ms, flip angle = 8^o^, isotropic resolution of 1mm^3^. Functional MRI was obtained by multiband echo-plannar imaging (EPI, University of Minnesota sequence) to acquire blood-oxygen-level-dependent (BOLD) sensitive images: TR = 2000 ms; TE = 30 ms; flip angle = 82°; IPAT = 2, multiband factor = 2, isotropic resolution of 2mm^3^ and 66 axial slices 2 mm thick (no gap) to ensure full brain coverage. Additionally, field-maps were acquired using two opposite coded (AP, PA) echo-plannar scans: TR = 8152 ms; TE = 66 ms; flip angle = 90°; multiband factor = 1; isotropic resolution of 2mm^3^ and 66 axial slices 2 mm thick (no gap). All images were acquired in the anterior commissure – posterior commissure (AC–PC) line extending down from the top of the brain.

Functional images were pre-processed using the FMRIB Software Library, version 6.0.0 (Oxford University, Oxford, United Kingdom, http://www.fmrib.ox.ac.uk/fsl) (Jenkinson, Beckmann, Behrens, Woolrich, & Smith, 2012). The following steps were conducted: removal of non-brain structures using BET (Smith, 2002); temporal high pass filtering with a cut off of 100s; fieldmap-based distortion-correction; rigid body motion correction, aligned to middle volume as an initial template image, using MCFLIRT (Jenkinson, Bannister, Brady, & Smith, 2002); spatial smoothing with a 5 mm isotropic Gaussian kernel. To normalize images to the Montreal Neurological Institute (MNI) template, low-resolution EPI images were first registered to higher-resolution EPI reference images (expanded functional images), then to a high resolution T1 images (using a linear, normal search registration) (Jenkinson et al., 2002; Jenkinson & Smith, 2001), then to the 152T1 MNI template (using a non-linear registration) (Andersson, Jenkinson, & Smith, 2007).

To reduce artifacts, Independent Component Analysis (ICA) was performed using Multivariate Exploratory Linear Optimized Decomposition into Independent Components (MELODIC). Components from ten participants were hand classified as ‘noise’ or ‘signal’ (Griffanti et al., 2017). Then, using this hand-classified training dataset, data was cleaned with FMRIB's ICA-based Xnoiseifier (FIX, version 1.066). This procedure also included motion confounds cleanup with highpass filtering.

An event-related design was used for the task. 500 ms events (‘attend toward threat’, ‘attend toward neutral’, ‘attend away from threat’, ‘attend away from neutral’) were convolved with a canonical double-gamma HRF to generate the model regressors.

### Social anxiety

The primary clinical outcome was total score on the clinician-administered Liebowitz Social Anxiety Scale (LSAS) (Liebowitz, 1987). Cronbach's alphas in the current study were 0.86 and 0.92 at pre- and post-treatment, respectively.

### General procedure

Following a baseline clinical evaluation, participants completed an MRI session consisting of structural, resting state, and the attention task. Then, participants were randomly assigned to 12 weeks of SSRI, GC-MRT, or WL. In week 13, a clinical evaluation and a second MRI session with the same parameters as the baseline session took place. The study was conducted from July 2018 to December 2021.

### Statistical analyses

Clinical effects were tested using a repeated-measures analysis of variance (ANOVA) with group (GC-MRT, SSRI, WL control) as a between-subjects factor and time (baseline, post-treatment) as a within-subject factor. Significant ANOVA results were followed by pairwise corrected independent samples *t* tests.

The effects of group, time, congruency with GC-MRT training, and group-by-time-by-congruency interaction on task accuracy and reaction-time (RT), were tested using repeated-measures ANOVAs with group (GC-MRT, SSRI, WL control) as a between-subjects factor and time (baseline, post-treatment) and congruency (incongruent trials, congruent trials) as within-subject factors. The task was designed to be behaviorally insensitive (Browning et al., 2010) and therefore allowed interpretation of the imaging data not confounded by behavioral differences between groups or time-points.

Imaging data analyses used FMRIB Software Library (FSL), version 6.0.0 (Oxford University, Oxford, United Kingdom, http://www.fmrib.ox.ac.uk/fsl) (Jenkinson et al., 2012). Incongruent>congruent blood-oxygen-level-dependent (BOLD) signal contrast maps were computed: training-incongruent trials were ‘attend face’ trials with disgusted faces and ‘attend bars’ trials with neutral faces, and training-congruent trials were ‘attend face’ trials with neutral faces and ‘attend bars’ trials with disgusted faces. This contrast probed specific alterations in threat-related attentional processes – the target of GC-MRT. Specifically, following the logic of Browning et al. (2010), the selected contrast enabled testing neural activation associated with attention allocation patterns that are opposite to the pattern to which GC-MRT patients were trained (Browning et al., 2010), and presumably requiring greater neural recruitment to oppose. Contrast images were calculated individually for each participant in each run, and then collapsed across all four runs to generate post>pre contrast maps.

The statistical analyses used to test hypotheses 1 and 2 were modeled after a recently published paper by Goldin et al. (2021), using a similar design to address similar questions. To test hypothesis 1, treated patients (GC-MRT/SSRI) were compared to WL participants on the post>pre contrast maps. To test hypothesis 2, GC-MRT patients were compared to SSRI patients on the post>pre contrast maps. For hypothesis 3, the correlations between pre-to-post clinical change (total LSAS difference score) and pre-to-post activation change (incongruent>congruent contrast difference maps) were tested. Although our hypotheses suggest potential effects in specific brain regions, given the scarcity of evidence on neural changes following GC-MRT and the possibility that this new treatment may affect additional unpredicted brain regions, we opted to apply a conservative whole-brain analytic approach and bidirectional hypotheses testing. Of note, although we defined the post>pre as the positive contrast when computing brain maps, the opposite direction of change, i.e., pre>post contrast, could also be represented in clusters with negative *z* values. This way, both contrasts were tested, and a bidirectional approach was maintained both for the group and time variables. Whole brain analyses were corrected for multiple comparisons using parametric cluster-based correction. A cluster forming threshold of *z* > 2.3 was applied at the voxel level, followed by an FWE correction at a significance level of *p* < 0.05 that was applied to the resulting clusters. This approach, combining voxel-level thresholding and FWE correction, is widely used in fMRI studies. It is also the default correction approach in FSL. Similar thresholds for cluster forming and FWE correction (*z* > 2.3; *p* < 0.05) have been applied in a number of fMRI studies in psychopathology (e.g. Aghajani et al., 2016; Foerde, Steinglass, Shohamy, & Walsh, 2015; Walsh et al., 2019; White et al., 2013), and specifically by Browning et al. (2010) from whom the task used in the current study was adapted.

Two additional contrasts were used to explore changes in neural response to threat during direct (i.e. when patients are instructed to process the faces) or indirect (i.e. when patients are instructed to process the bars) face processing. Threat *v.* neutral contrasts were computed separately for ‘attend face’ trials (i.e. contrasting toward threat trials *v.* toward neutral trials) and ‘attend bars’ trials (i.e. contrasting away from threat trials *v.* away from neutral trials). An additional contrast, toward neutral *v.* away from threat, was also tested. Group comparisons (treated patients *v.* WL patients and GC-MRT *v.* SSRI patients) of pre-to-post changes were performed using these separate contrasts as described for the ‘incongruent *v.* congruent’ contrast.

Between-group and between-time differences in framewise displacement were tested to verify that significant effects emerged from the above-mentioned analyses were not related to head motion differences (see online Supplementary Material). Localization of significant clusters was based on peak coordinates reported in previous studies. For further specification, distinct clusters within the ACC were numbered from rostral (specified as ‘ACC_1_’) to caudal (specified as ‘ACC_3_’).

To interpret significant results and explore significance and direction of change within each group and time-point, post-hoc analyses were performed using the average percent signal change within each significant cluster (Anticevic et al., 2014; Su et al., 2016). For hypotheses 1 and 2, these analyses were performed as follows: First, simple effects of group and time were tested using independent samples *t* tests and paired samples *t* tests, respectively. For completeness, exploratory time-simple-effect tests for each of the treatment groups (GC-MRT, SSRI) were performed for the clusters extracted from the treatment *v.* WL main analysis; and a time-simple-effect was tested within the WL group for the cluster extracted from the GC-MRT *v.* SSRI main analysis. Finally, for clusters in which there were significant baseline between-group differences in mean BOLD signal change, we conducted additional analyses controlling for baseline BOLD signal change as a covariate; these tests for baseline group differences and subsequent covariate analyses are described in the online Supplementary Material. For hypothesis 3, exploratory post-hoc analysis included averaging the percent signal change within the examined cluster both at baseline and at post-measurement, then calculating the difference between the two time-points for each participant. A simple correlation coefficient was used to test the association between this activation change and change in total LSAS scores. Fisher's *r*-to-*Z* transformations were used to compare the magnitude of these correlations between groups. To test whether activation-symptom correlation specifically characterized brain regions that emerged as sensitive to intervention in the current study, we also applied this analysis using the mean activation change within the significant clusters that emerged from analyses performed for hypotheses 1 and 2. All these follow-up analyses were conducted using IBM SPSS Statistics, version 28.0.

## Results

Data analysis included 81 patients (GC-MRT: *n* = 29; SSRI: *n* = 22; WL: *n* = 30).

### Demographics and clinical outcomes

Clinical outcomes in MRI-completers resembled those noted in the full sample (Arad et al., 2023). This involved a significant Time-by-Group interaction (*F*[2, 78] = 10.00, 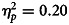 [90% CI 0.07 to 0.32]). Follow-up analyses indicated that both GC-MRT and SSRI were associated with lower symptoms post-treatment compared to the WL control group (GC-MRT: *t*[57] = −3.56. *p* < 0.001, *d* = −0.93 [95% CI −1.46 to −0.38]; SSRI: *t*[50] = −3.49. *p* < 0.001, *d* = −0.98 [95% CI −1.56 to −0.39]). No significant difference in LSAS scores was noted between GC-MRT and SSRI post-treatment (*p* = 0.95). For detailed clinical results of the full sample, including Clinically Significant Change and Reliable Change, see Arad et al. (2023). Also see Arad et al. (2023) for a description of cognitive target engagement (i.e. changes in dwell time on threat faces) among GC-MRT patients.

## Neuroimaging

### Task behavioral performance

Per design, the attentional task was behaviorally insensitive (Browning et al., 2010) with high accuracy at pre- (*M* = 91%, s.d. = 8%) and post-treatment (*M* = 92%, s.d. = 9%). There were no significant effects of time, group, congruency, or time-by-group-by-congruency interaction on accuracy (*p*s> 0 .22). As expected, there was a significant main effect of time on RT, with lower RTs at post-treatment scans compared to baseline scans (*F*[1, 78] = 23.43, 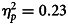 [90% CI 0.10 to 0.35]). There were no significant effects of group, congruency, or time-by-group-by-congruency interaction on RT (*p*s > 0.17). For additional analyses of behavioral performance conducted separately for all four task conditions, see online Supplementary Material.

### Treatments *v.* waitlist control (hypothesis 1)

**Figure 2.**
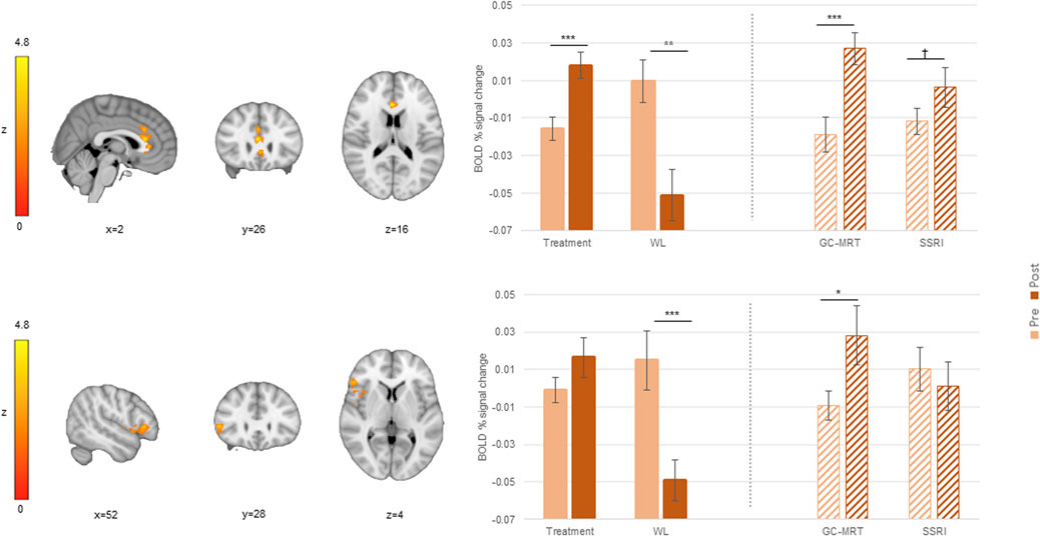
ACC_2_ and rIFG clusters in which pre-to-post increase in BOLD signal change (incongruent>congruent contrast) was different between treatment (GC-MRT and SSRI) and WL patients; and the mean BOLD signal change within every significant cluster, by group and time. Separate post-hoc analyses for GC-MRT and SSRI patients are also presented (striped bars). For simplicity, only time simple-effects significance is labeled (*** *p* < 0.001; ** *p* < 0.01; *p < 0.05; † *p* = 0.056). BOLD, blood-oxygen-level-dependent; GC-MRT, gaze contingent music reward therapy; SSRI, serotonin reuptake inhibitors; WL, waitlist. Error bars represent standard errors.

When required to direct attention contrary to GC-MRT training (incongruent>congruent contrast), treated patients showed greater pre-to-post increase in BOLD signal change in ACC_2_ (one of the two more rostral of the significant ACC clusters) and the right inferior frontal gyrus (rIFG), compared to WL participants (Table 1 and Fig. 2). In ACC_2_, a significant activation increase was noted among treated patients (*t*[50] = −4.34, *p* < 0.001, *d* = −0.61 [95% CI −0.90 to −0.31]) whereas WL patients showed a significant decrease in activation (*t*[29] = 3.36, *p* = .002, *d* = 0.61 [95% CI 0.22 to 1.00]). In the rIFG, WL participants showed an activation decrease (*t*[29] = 4.19, *p* < 0.001, *d* = 0.76 [95% CI 0.35 to 1.17) whereas treated patients showed no change in activation (*t*[50] = −1.59, *p* = 0.12, *d* = −0.22 [95% CI −0.50 to 0.06]).

**Table 1.** Significant clusters emerged for the incongruent>congruent contrast and the attend-threat>attend-neutral contrast (direct threat processing), in group comparisons of pre-to-post BOLD activation increase and correlation tests of clinical improvement and pre-to-post BOLD activation increase association

Contrast	Analysis		Region	MNI coordinates	Voxels	*Z*-score (peak)
Incongruent to training > Congruent to training	Between-group comparisons	Treatment *v.* WL	ACC_2_	2 26 16	245	4.62
			rIFG	52 28 4	263	3.68
		GC-MRT *v.* SSRI	ACC_3_	2 8 28	259	3.67
	Correlation with clinical improvement (GC-MRT)		dlPFC	30 50 20	346	3.70
Attend threat > Attend neutral	Between-group comparisons	Treatment *v.* WL	ACC_1_	2 32 18	1152	4.12

BOLD, blood-oxygen-level-dependent; WL, waitlist; GC-MRT, gaze contingent music reward therapy; SSRI, serotonin reuptake inhibitors; ACC, anterior cingulate cortex; rIFG, right inferior frontal gyrus; dlPFC, dorsolateral prefrontal cortex; MNI, Montreal neurological institute.

Clusters within the ACC are numbered from rostral (ACC_1_) to caudal (ACC_3_).

Simple effects analyses performed separately for the two treatment groups revealed that for both ACC_2_ and rIFG clusters, activation increased in the GC-MRT group (ACC_2_: *t*[28] = −3.96, *p* < 0.001, *d* = −0.73 [95% CI −1.14 to −0.32]; rIFG: *t*[28] = −2.35, *p* = 0.03, *d* = −0.44 [95% CI −0.81 to −0.05]). For SSRI patients, ACC_2_ activation increase was marginally significant (*t*[21]= −2.02, *p* = 0.056, *d* = −0.43 [95% CI −0.86 to 0.11]), and rIFG activation did not significantly change (*t*[21] = 0.75, *p* = 0.46, *d* = 0.16 [95% CI −0.26 to 0.58]) (Fig. 2).

### GC-MRT *v.* SSRI (hypothesis 2)

**Figure 3.**
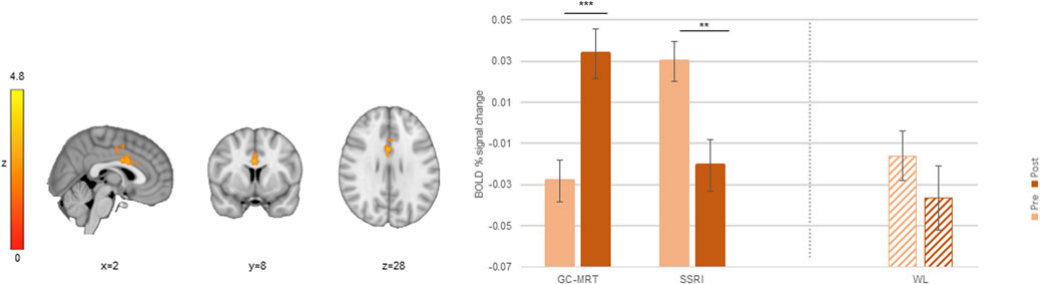
An ACC_3_ cluster in which pre-to-post increase in BOLD signal change (incongruent>congruent contrast) was different between GC-MRT and SSRI patients; and the mean BOLD signal change within this significant cluster, by group and time. Post-hoc analysis within this region is also presented for WL participants (striped bars). For simplicity, only time simple-effects significance is labeled (*** *p* < 0.001; ** *p* < 0.01). BOLD, blood-oxygen-level-dependent; GC-MRT, gaze contingent music reward therapy; SSRI, serotonin reuptake inhibitors; WL, waitlist. Error bars represent standard errors.

Compared to SSRI patients, GC-MRT patients showed a greater pre-to-post increase in incongruent>congruent signal change in ACC_3_ (the more caudal of the significant ACC clusters; see Table 1 and Fig. 3). Follow-up analyses indicated a significant pre-to-post increase in neural activation within this cluster among GC-MRT patients (*t*[28] = −4.24, *p* < 0.001, *d* = −0.79 [95% CI −1.20 to −0.36), whereas the opposite pattern emerged in SSRI patients (*t*[21] = 3.61, *p* = 0.002, *d* = 0.77 [95% CI 0.28 to 1.24]).

Applying a simple effect analysis on the same cluster in the WL control group indicated no significant pre-to-post change in mean signal change (*t*[29] = 1.07, *p* = 0.29, *d* = 0.19 [95% CI −0.17 to 0.55]) (Fig. 3).

### Neural change – clinical change association (hypothesis 3)

**Figure 4.**
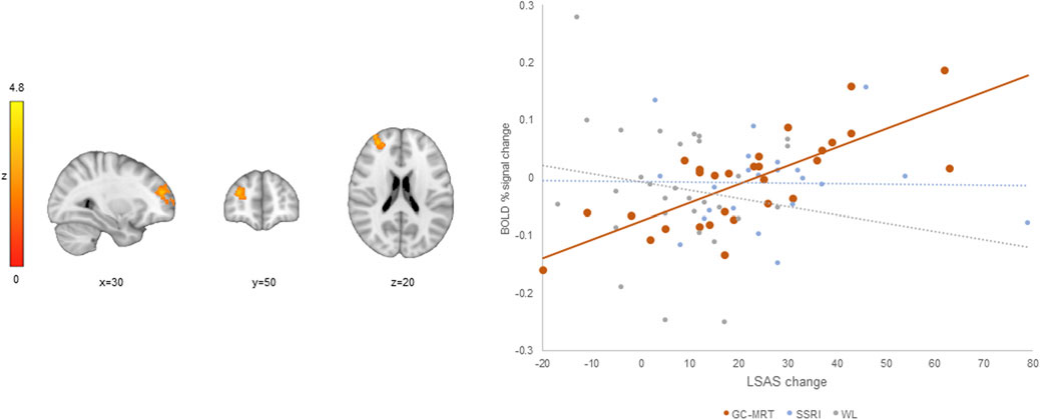
A dlPFC cluster in which pre-to-post increase in BOLD signal change (incongruent>congruent contrast) was significantly correlated with clinical improvement among GC-MRT patients. The scatter plot presents the correlation between the mean BOLD signal change within this cluster and the clinical change (GC-MRT: *r* = 0.75, *p* < 0.001, *R^2^* = 0.56; SSRI and WL: *p*s > 0.39). BOLD, blood-oxygen-level-dependent; GC-MRT, gaze contingent music reward therapy.

Whole brain analysis revealed that in the GC-MRT group, a positive correlation was noted between pre-to-post symptom change and incongruent>congruent activation change, within a single cluster in the dorsolateral prefrontal cortex (dlPFC) (see Table 1 and Fig. 4). Follow-up analysis indicated a strong correlation between symptom change and this cluster's averaged activation change (*r* = 0.75, *p* < 0.001, *R^2^* = 0.56 [95% CI 0.34 to 0.78]) (Fig. 4). For the SSRI and WL groups, the correlations between symptom change and averaged activation change within this cluster were not significant (all *p*s > 0.39); *r*-to-*Z* transformations showed that the observed correlation in the GC-MRT group was greater than the correlations in the SSRI and WL groups (*r*-to-*Zs* = 3.29 and 4.13, *ps* < 0.001, respectively). No significant clusters emerged for clinical and neural change correlations in the SSRI or WL groups. No significant correlations were found between symptom change and activation change within brain regions in which significant clusters were found in hypothesis 1 and 2 (See online Supplemental Table S4).

### Direct and indirect threat processing

**Figure 5.**
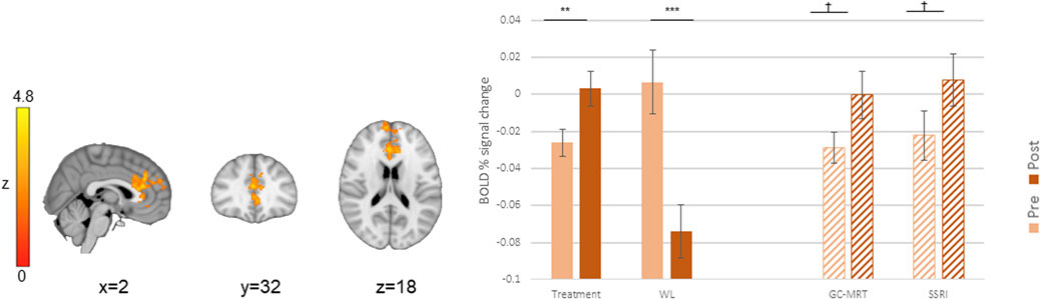
An ACC_1_ cluster in which pre-to-post increase in BOLD signal change during direct threat processing (toward threat > toward neutral) was different between treatment (GC-MRT and SSRI) and WL patients; and the mean BOLD signal change within this cluster, by group and time. Separate post-hoc analyses for GC-MRT and SSRI patients are also presented (striped bars). For simplicity, only time simple-effects significance is labeled (*** *p* < 0.001; ** *p* < 0.01; GC-MRT: † *p* = 0.068; SSRI: † *p* = 0.073). BOLD, blood-oxygen-level-dependent; GC-MRT, gaze contingent music reward therapy; SSRI, serotonin reuptake inhibitors; WL, waitlist. Error bars represent standard errors.

Treated patients showed greater pre-to-post increase in BOLD signal change in ACC_1_ compared to WL participants (Table 1 and Fig. 5) during direct processing of threat faces. In ACC_1_, a significant increase in attend threat > attend neutral activation increase was noted among treated patients (*t*[50] = −2.69, *p* < 0.01, *d* = −0.38 [95% CI −0.66 to −0.09]) whereas WL participants showed a significant decrease in activation (*t*[29] = 4.85, *p* < 0.001, *d* = 0.88 [95% CI 0.46 to 1.30). A separate simple effects analyses for the two treatment groups showed a non-significant trend level increase in both GC-MRT and SSRI groups (GC-MRT: *t*[28] = −1.90, *p* = 0.068, *d* = −0.35 [95% CI −0.72 to 0.03]; SSRI: *t*[21] = −1.89, *p* = 0.073, *d* = −0.40 [95% CI −0.83 to 0.04]) (Fig. 5). In this brain region, there were no significant pre-to-post activation changes during indirect threat processing (attend threat > attend neutral contrast) (see online Supplement Fig. S5). No group differences were evident during indirect threat processing or for the toward neutral *v.* away from threat contrast.

## Discussion

We investigated common and specific effects of GC-MRT and SSRI treatments on brain function during social threat processing. Three key findings emerged: (a) activation changes in a rostral region of the ACC and in the rIFG differentiated between treated (GC-MRT/SSRI patients) and non-treated waitlist patients; (b) activation changes in a distinct, caudal cluster within the ACC, differentiated between GC-MRT and SSRI patients; and (c) GC-MRT patients showed a strong association between clinical improvement and dlPFC activation change when required to act against the action they were trained for in treatment (i.e. attend threat rather than neutral faces). Together, these results suggest both common and unique treatment effects of attention bias modification and SSRI on brain function.

Within the regions that differentiated between treated and non-treated patients (ACC_1_, ACC_2_, and rIFG), patients in the WL group exhibited decrease in activation in the second session, possibly reflecting habituation to the previously encountered stimuli. In contrast, activation in these areas increased significantly among treated patients, suggesting a treatment-related effect. The ACC has been associated with downregulation of emotional responses and mediation of cognitive influences on emotion (Bush, Luu, & Posner, 2000; Holroyd & Umemoto, 2016; Segalowitz & Dywan, 2008; Stevens, Hurley, & Taber, 2011). The current findings converge with previous reports on functional changes within these brain regions following clinically effective pharmacological and cognitive-behavioral interventions for SAD (Carlson et al., 2022; Giménez et al., 2014; Goldin, Manber, Hakimi, Canli, & Gross, 2009a; Goldin, Manber-Ball, Werner, Heimberg, & Gross, 2009b; Klumpp, Fitzgerald, & Phan, 2013a; Månsson et al., 2013). The ACC is a part of the salience network, a neural network attuned to the salience of external and internal stimuli (Seeley, 2019; Seeley et al., 2007). The salience network is also specifically involved in detecting changes in facial emotion expressions (Luo et al., 2014; Rosen et al., 2018). Irregular function of the salience network was reported in patients with SAD (see: Kim and Yoon, 2018). Therefore, the current findings may represent treatment-related changes in salience network function.

The rIFG has been associated with inhibitory control, including the intention to stop or slow action, or to suppress an urge for action (Aron, Robbins, & Poldrack, 2004, 2014; Rubia, Smith, Brammer, & Taylor, 2003; Shadli et al., 2020; Verbruggen & Logan, 2008); this may suggest that the two active treatments for SAD applied in the current study are related to enhanced recruitment of inhibitory brain functions when patients are instructed to divert their attention away from neutral stimuli and toward threatening ones. Such change in inhibitory functions may be particularly relevant for patients in the GC-MRT group, due to the requirement in the applied task to respond contrary to the pattern they had practiced repeatedly during treatment. Considering that the interaction effect for the rIFG was partially driven by a decrease in activation in the WL group, it may be argued that these changes are also related to habituation to the task in this group. According to this interpretation, it is possible that treatment countered this basic habituation effect. Notably, follow-up analyses indeed suggest that whereas GC-MRT patients show significant increases in both ACC_2_ and rIFG activations from pre- to post-treatment, SSRI patients showed only a trend level increase in ACC_2_ and a non-significant change in rIFG. The similar pattern of treatment-related ACC_2_ activation increase in both treated groups potentially reflect a change in general threat monitoring mechanisms tapped into by both the GC-MRT (see results from Browning et al. (2010)) and SSRI protocols, and that the rIFG may be more specifically affected by GC-MRT than by SSRI. This interpretation is also in line with the results of the exploratory analysis indicating a similar pattern of change in a close and overlapping region of the rostral ACC (ACC_1_) during direct, but not indirect, threat processing. Thus, the noted activation changes in the rostral ACC could reflect changes in a more general response to direct threat processing following treatment, that potentially drives the findings that emerged in ACC_2_ in training incongruent *v.* congruent activation.

Importantly, the direct comparison between the two active treatments in the current study revealed treatment-specific changes. Patients receiving GC-MRT showed pre- to post-treatment increase in ACC_3_ activation, whereas the opposite pattern was noted for SSRI patients. Post-hoc analyses further indicate that within this specific cluster, activation among WL participants did not significantly change and was similar in pattern to that of the SSRI group. While the cluster differentiating between treated to non-treated patients in general was located more rostral within the ACC (ACC_2_), the cluster distinguishing pre-to-post treatment changes between GC-MRT and SSRI was found more caudal (ACC_3_). The rostral section of the ACC is often described as the ‘affective’ ACC division, and has been associated with emotional conflict and emotion suppression in healthy individuals, and with symptom provocation in anxiety (Bush et al., 2000; Drevets & Raichle, 1998; Etkin, Egner, Peraza, Kandel, & Hirsch, 2006; Polli et al., 2005). The caudal ACC is typically considered as the ‘cognitive’ sub-region, reported to be activated when reduction of cognitive conflict is required in divided-attention and working-memory tasks (Bush et al., 2000; Drevets & Raichle, 1998; Kerns et al., 2004; Polli et al., 2005). It is conceivable that GC-MRT patients, who deliberately practiced diverting their gaze away from threat faces and toward neutral faces, needed to recruit more caudal-ACC resources when faced with the opposite requirement in the fMRI task (i.e. attending toward threatening faces and away from neutral faces).

Moreover, GC-MRT patients showed a strong association between clinical improvement and dlPFC activation change when faced with this requirement. These findings join the results of an earlier study (Browning et al., 2010) using the same task, in which hyperactivation of the dlPFC was noted among individuals who were requested to act contrary to the attentional pattern they had trained for in a manual reaction-time-based ABM intervention (Browning et al., 2010). Of note, the dlPFC is also involved in emotion categorization (Cacioppo, Crites, Berntson, & Coles, 1993; Freedman, Riesenhuber, Poggio, & Miller, 2003; Zwanzger et al., 2014). It is possible that GC-MRT uniquely affected dlPFC function leading to the use of a different categorization strategy during the attention task. This difference could be manifested in the noted association between symptom change and dlPFC activation during task performance. Although the findings in Browning et al., (2010) suggest that the dlPFC may be sensitive to cognitive changes following ABM, interpreting the activation-symptom correlation found in the dlPFC in the current study may be challenging, considering that this brain region did not emerge as sensitive to shared (hypothesis 1) or distinct (hypothesis 2) treatment effects.

The current results also correspond with a study describing heightened error-related negativity (ERN) as a predictor of GC-MRT clinical efficacy for SAD (Umemoto et al., 2021). The ERN is thought to be generated in the caudal part of the ACC (Cavanagh & Shackman, 2015; Holroyd & Umemoto, 2016; Yeung, Botvinick, & Cohen, 2004). It is therefore possible that GC-MRT specifically elevates threat-related attention processing in this brain region assisting patients with SAD to down-regulate their predisposed hypersensitivity to social threats, gain better ability to disengage from such threats, and subsequently experience relief in SAD symptoms.

A few limitations of the current study should be noted. First, although the use of a waitlist control affords an important read of the effects of repeated assessments on brain function, this condition limits the capacity to differentiate specific neural effects of active treatment components from those of non-specific treatment effects. Future studies on the neural correlates of these treatments may wish to test their effects on brain function in comparison to placebo pills and a sham computerized training control, for the SSRI and GC-MRT treatments, respectively (Lazarov et al., 2017). Future studies may also wish to compare GC-MRT to CBT. Whereas we decided to contrast GC-MRT with a treatment that potentially relies on different mechanisms of actions, it may also be valuable to examine whether GC-MRT and CBT – both targeting cognitive processes and attentional patterns specifically – have common neural mechanisms. Second, differential drop-out rates between the groups may affect the generalizability of the current findings; specifically, the higher drop-out in the SSRI group could have affected the characteristics of the final sub-sample on which analyses were performed. Third, fMRI scans took place at baseline and post-treatment. An addition of interim mid-treatment scans could shed additional light on potential between-treatments differences in the trajectories of neural changes over time. However, it is worth noting that the clinical effect of GC-MRT has been shown to last at least three months (Lazarov et al., 2017), and that the rate of clinical improvement over time in the current sample closely overlapped in GC-MRT and SSRI treatment (see: Arad et al., 2023). Fourth, the current study was not powered to test for all possible main and interaction effects using an omnibus whole-brain mixed ANOVA. Future studies could use larger samples to enable such an analysis. Fifth, the currently reported decrease in activation among WL patients, interpreted here as reflecting a habituation effect, may suggest that the attention task has low test-retest reliability. Although the accuracy and RT measures derived from the task indicate adequate reliability, correlations between pre- and post-scans in WL patients suggest low reliability. Unfortunately, low test-retest reliability often characterizes fMRI tasks, and call for the development of more stable and reliable measures (Bennett & Miller, 2010; Elliott et al., 2020; Noble, Scheinost, & Constable, 2019, 2021). Future studies may wish to test long-term functional changes of GC-MRT using additional follow-up measurements. Previous findings indicate long-term clinical effects for GC-MRT (Lazarov et al., 2017), which could be mirrored by certain sustained neural effects over time. Lastly, considering that SAD has been associated with aberrant patterns of neural connectivity during social threat processing (Evans, Bar-Haim, Fox, Pine, & Britton, 2020; Gold et al., 2016; Gorka et al., 2015; Pantazatos et al., 2014; Sequeira et al., 2021), future studies could extend the current findings by testing whether GC-MRT induces changes in the functional associations between different brain regions. Such studies may focus on the connectivity between the regions described here as functionally changing following GC-MRT, their connectivity with the limbic system (Brühl et al., 2014; Etkin & Wager, 2007; Sylvester et al., 2012; Xu et al., 2019), and the relations between these connectivity patterns and SAD symptom change.

In conclusion, the current study shows that both GC-MRT and SSRI are effective treatments for SAD and lead to changes in brain function during implicit social threat processing. These changes take place in brain regions associated with attention control and inhibitory functions, supporting the role of such basic cognitive processes in the maintenance of SAD symptoms. The results also highlight the potential of targeting these neuro-cognitive processes through focused therapeutic interventions. The results further suggest treatment-specific neural pathways of clinical change in the ACC and dlPFC, suggesting that patients with SAD who do not fully benefit from SSRIs or GC-MRT may potentially benefit from the alternative treatment, or from a combination of the two.

## Supporting information

Azriel et al. supplementary material Azriel et al. supplementary material

## Supplementary material

The supplementary material for this article can be found at https://doi.org/10.1017/S0033291724001521.
