# Supplementary material for: Neural activation changes following attention bias modification treatment or a selective serotonin reuptake inhibitor for social anxiety disorder
Source: Psychol Med. 2024 Sep 10;54(12):3366–78. doi: 10.1017/S0033291724001521 (PMC11496228; doi:10.1017/S0033291724001521)
Supplement: Azriel et al. supplementary material [file S0033291724001521sup001.docx]

**Neural Activation Changes following Attention Bias Modification Treatment or a Selective Serotonin Reuptake Inhibitor for Social Anxiety Disorder**

***Supplementary Material***

Pre-Treatment Neural Response (Manipulation Check): Page 3

Supplementary Table S1: Page 5

Supplementary Figure S1: Page 7

Supplementary Figure S2: Page 8

Supplementary Figure S3: Page 9

Attention Task Test-Retest Reliability: Page 10

Framewise Displacement (Head Motion) Analysis: Page 11

Attention Task Performance – Separate Analyses for Four Task Conditions: Page 12

Supplementary Table S2: Page 13

Baseline Group Differences in Significant Clusters and Covariate Analyses: Page 14

Supplementary Table S3: Page 15

Supplementary Figure S4: Page 16

Supplementary Table S4: Page 17

Supplementary Figure S5: Page 18

Significant Clusters Localization: Page 19

References: Page 20

**Pre-Treatment Neural Response (Manipulation Check)**

A repeated-measures analysis of variance (ANOVA) with attention (toward face, away from face) and valence (threat, neutral) as between-subjects factors was performed on 97 baseline scans, corrected for multiple comparisons using cluster forming threshold of z=2.3 and familywise error correction (FWE) at a level of p<.05.

This analysis indicated a significant main effect for attention in a neural network consisting of eight clusters (Table S1). Activation reflecting toward face > away from face was found in the fusiform face area (FFA) and prefrontal cortex (PFC), whereas activation reflecting away from face > toward face was found in several regions comprising the dorsal attention network (DAN) (Yeo et al., 2011) (Figures S1 and S3). The FFA and PFC activations found here converge with previous evidence on brain regions activated during face processing (Kanwisher & Yovel, 2006; Marinkovic, Trebon, Chauvel, & Halgren, 2000). The DAN activation presented during trials in which attention was oriented away from a previous fixation location and a potentially distracting face, is in line with existing evidence on the role of the DAN in voluntary orienting of visuo-spatial attention (Kincade, Abrams, Astafiev, Shulman, & Corbetta, 2005; Lanssens, Pizzamiglio, Mantini, & Gillebert, 2020).

A significant main effect for valence was found in a neural network comprised of five clusters (Table S1). Activation reflecting threat > neutral was found in the FFA, whereas activation reflecting neutral > threat was found in regions comprising the visual network (VN) and the somatomotor network (SMN) (Yeo et al., 2011) (Figures S2 and S3). Previous reports suggest that threat affects FFA activation (Beaton et al., 2009; Goldin, Manber, Hakimi, Canli, & Gross, 2009; Kret, Pichon, Grèzes, & De Gelder, 2011; Monroe et al., 2013). The greater activation of the VN and SMN may be related to patients’ reduced avoidance in the presence of neutral compared to threat faces (Staugaard, 2010), resulting in greater visual and motor processing required for task performance.

No significant clusters were found for the attention-by-valence interaction effect, and no significant differences were found between treatment completers and non-completers for these pre-treatment neural responses.

**Table S1.** Significant clusters emerged for ANOVA main effect of attention (toward face, away from face) and valence (threat, neutral) at pre-treatment. DAN, dorsal attention network; PFC, prefrontal cortex; FFA, fusiform face area; VN, visual network; SMN, somatomotor network; MNI, Montreal neurological institute.

| **Effect** | **Region** | **MNI coordinates** | **Voxels** | **Z-score (peak)** |
| --- | --- | --- | --- | --- |
| Attention | DAN | -54 -62 -6 | 20684 | 7.91 |
|  | DAN | -28 -10 50 | 987 | 4.93 |
|  | DAN | 28 -4 50 | 960 | 5.47 |
|  | DAN | -50 6 34 | 725 | 6.59 |
|  | DAN | 52 6 26 | 565 | 9.06 |
|  | PFC | -6 58 28 | 536 | 4.24 |
|  | FFA | 38 -48 -20 | 416 | 5.19 |
|  | PFC | 36 62 10 | 248 | 4.23 |
| Valence | VN | 24 -86 -10 | 15133 | 6.71 |
|  | SMN | 36 -26 68 | 5308 | 5.33 |
|  | SMN | -66 -16 6 | 2344 | 5.51 |
|  | SMN | 64 -22 12 | 1562 | 5.6 |
|  | FFA | 46 -40 -20 | 260 | 4.65 |

**Figure S1.** Brain regions in which there was a main effect for attention at baseline. Warm colours represent regions where away face > toward face, and cold colours represent regions where toward face > away face.


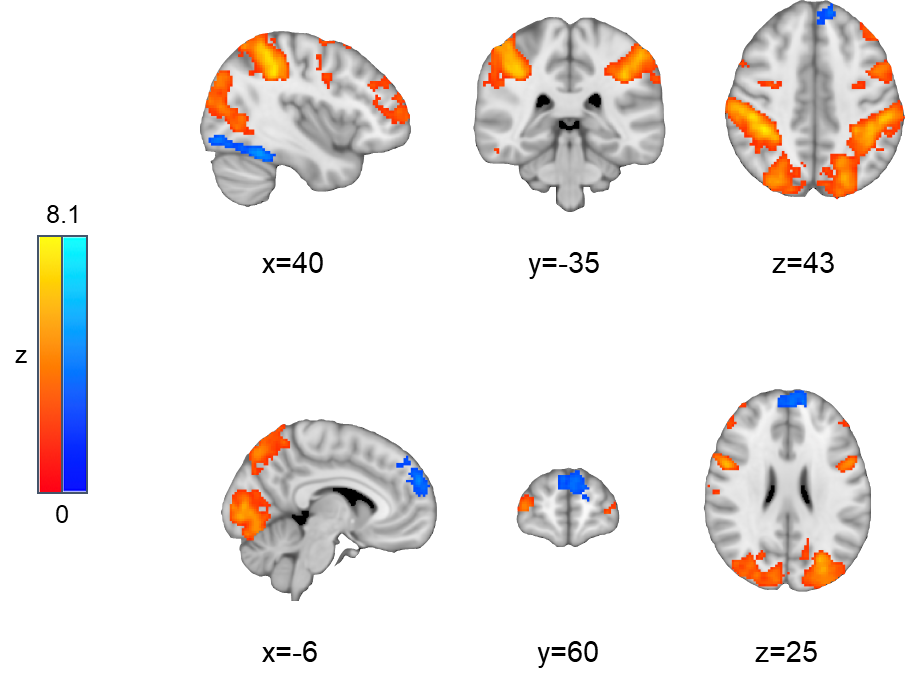


**Figure S2.** Brain regions in which there was a main effect for face valence at baseline. Warm colors represent regions where neutral > threat, and cold colors represent regions where threat > neutral.


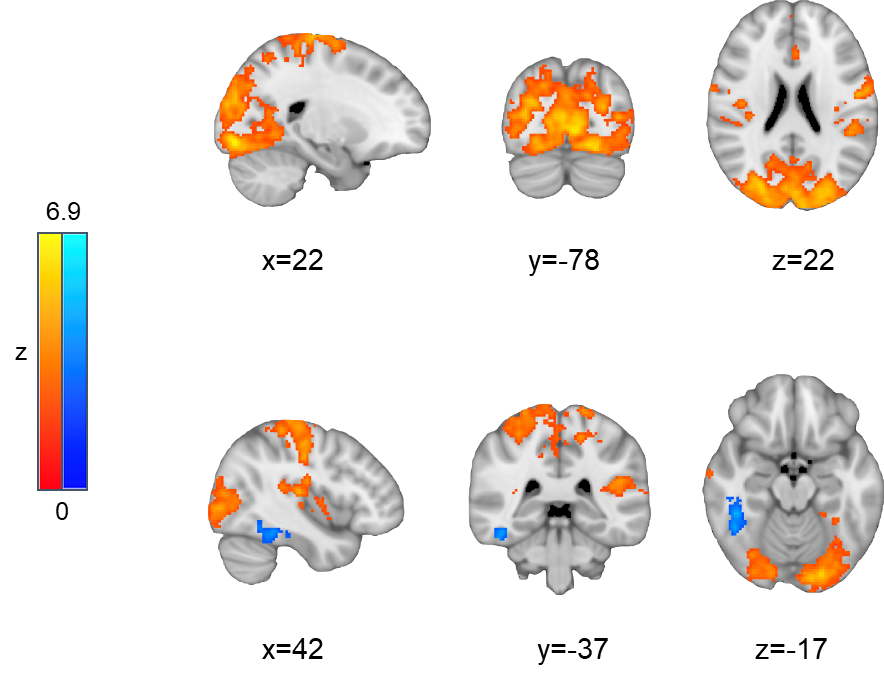


**Figure S3.** Overlap between brain regions with a significant main effect for attention and the DAN (A); and overlap between brain regions with a significant main effect for face valence and the VN / SMN (B). DAN, dorsal attention network; PFC, prefrontal cortex; FFA, fusiform face area; VN, visual network; SMN, somatomotor network.


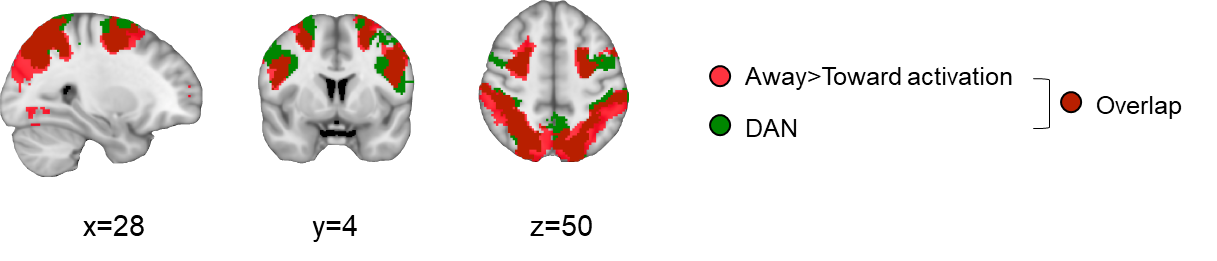


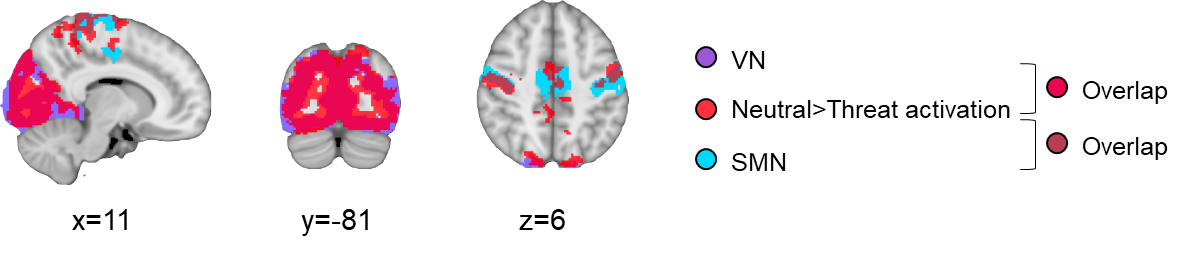


**Attention Task Test-Retest Reliability**

**Behavioral data.**

Pearson correlation coefficients were calculated between pre-treatment and post-treatment scan, and for accuracy and reaction-time (RT) measures. Since the purpose of this analysis was to assess test-retest reliability, only WL control patients (n=30) were included. Results indicated good test-retest reliability for these measures, with *r=*.73 (*p*<.001) for accuracy and *r=*.75 (*p*<.001) for RT.

**Imaging data.**

Intraclass correlation coefficients (ICC (C,k)) were calculated between the pre-treatment and post-treatment scans of WL patients, using BOLD signal change of the significant effects that emerged from the baseline manipulation check (See Table S1 and Figures S1 and S2). For each of these effects, a mask based on the relevant cluster was used. These analyses indicated low test-retest reliability for the Attention effect (ICC=.20) and the Valence effect (ICC=.05).

**Framewise Displacement (Head Motion) Analysis**

Framewise displacement (FD), computed with FSL motion outliers tool (Oxford University, Oxford, United Kingdom, <http://www.fmrib.ox.ac.uk/fsl>) (Jenkinson, Beckmann, Behrens, Woolrich, & Smith, 2012), was used to test for between-group and between-time differences in head motion. For each participant, FD was averaged across all four runs. Using this measure as a dependent variable, we conducted an ANOVA with group (GC-MRT, SSRI, WL control) as a between-subjects factor and time (baseline, post-treatment) as a within-subject factor. Results indicated no significant effects of group, time, or group-by-time interaction, on FD (*p*s>.225).

**Attention Task Performance – Separate Analyses for Four Task Conditions**

In addition to the main analysis conducted for the behavioral data, four separate analyses for each of the task conditions were performed. For each condition, the effects of group, time, and group-by-time interaction on task accuracy and reaction-time (RT), were tested using repeated-measures ANOVAs with group (GC-MRT, SSRI, WL control) as a between-subjects factor and time (baseline, post-treatment) as a within-subject factor.

The results of these analyses are presented in Table S2. In all four conditions, there was a significant main effect of time on RT, with faster RTs at post-treatment scans compared to baseline scans. A main effect of time on accuracy was found only in the away from threat and away from neutral conditions. In these two conditions, accuracy increased from pre- to post-treatment. Interestingly, as reported in the main text, a significant effect of time on RT was evident also in the incongruent condition (a combination of toward threat and away from neutral conditions) and the congruent condition (a combination of toward neutral and away from threat conditions), whereas no effect of time on accuracy was found in these two combined conditions. While RT and accuracy do not necessarily reflect the same processes and could offer distinct patterns in relation to task categories, further research could examine why such an improvement in accuracy occurred specifically when responding to bars rather than faces was required.

**Table S2.** Results of ANOVAs performed on accuracy and RT measures separately for each of the attention task’s four conditions.

| **Measure** | **Effect** | **Toward threat** | **Toward neutral** | **Away from threat** | **Away from neutral** |
| --- | --- | --- | --- | --- | --- |
| Accuracy | Time | F(1,78)=0.42, *p*=.520 | F(1,78)=0.74, *p*=.391 | F(1,78)=16.03, *p*<.001 | F(1,78)=5.45, *p*=.022 |
|  | Group | F(2,78)=1.02, *p*=.363 | F(2,78)=0.10, *p*=.902 | F(2,78)=0.31, *p*=.733 | F(2,78)=0.67, *p*=.516 |
|  | Time-by-group | F(2,78)=0.84, *p*=.437 | F(2,78)=0.41, *p*=.664 | F(2,78)=0.77, *p*=.467 | F(2,78)=1.13, *p*=.327 |
| Reaction Time (RT) | Time | F(1,78)=7.90, *p*=.006 | F(1,78)=8.03, *p*=.006 | F(1,78)=23.40, *p*<.001 | F(1,78)=15.65, *p*<.001 |
|  | Group | F(2,78)=0.135, *p*=.874 | F(2,78)=0.82, *p*=.921 | F(2,78)=0.84, *p*=.436 | F(2,78)=0.76, *p*=.470 |
|  | Time-by-group | F(2,78)=0.10, *p*=.900 | F(2,78)=0.74, *p*=.480 | F(2,78)=1.87, *p*=.161 | F(2,78)=1.15, *p*=.322 |

**Baseline Group Differences in Significant Clusters and Covariate Analyses**

Where between group differences in the mean BOLD signal change within a cluster were found at baseline, we conducted a follow up analysis controlling for baseline BOLD signal change as a covariate. To this end, we performed a univariate analysis of variance (ANOVA) with Group (treated and non-treated patients, or GC-MRT and SSRI) as an independent variable, pre to post increase in the mean % signal change as a dependent variable, and the baseline mean % signal change as a covariate.

Below we describe where baseline group differences were found, and the subsequent covariate analyses performed.

**Treatment vs. Waitlist control**

Between group difference in baseline signal change was noted for the ACC_1_ cluster (*t*(79)=-2.14, *p*=.03, *d*=-.49 [95%CI -0.95 to -0.03]), and controlling for baseline signal change the above-reported between group effects remained significant (*F*(1, 78)=26.29 , *p*<.001, $\eta_{p}^{2}$=.25 [90%CI 0.12 to 0.37]).

**GC-MRT vs. SSRI**

Between group difference in baseline signal change was noted for the ACC_2_ cluster (*t*(49)=-4.06, *p*<.001, *d*=-1.15 [95%CI -1.74 to -0.54]). Here too, when controlling for baseline BOLD signal change as a covariate, between group effects remained significant for this cluster (*F*(1, 48)=10.64, *p*=.002, $\eta_{p}^{2}$=.18 [90%CI 0.04 to 0.33]).

**Table S3.** Baseline demographic characteristics of analyzed participants, for each group. GC-MRT, Gaze Contingent Music Reward Therapy; SSRI, Selective Serotonin Reuptake Inhibitors; GAD, Generalized Anxiety Disorder.

|  | **GC-MRT (n=29)** | **SSRI (n=22)** | **Control (n=30)** |
| --- | --- | --- | --- |
| Males, no. (%) | 18 (62.07) | 13 (59.09) | 18 (60.0) |
| Age, mean (SD), years | 29.59 (7.03) | 28.82 (7.42) | 29.66 (7.06) |
| Comorbidities, no. (%) |  |  |  |
| Mild depressive episode | 7 (24.14) | 8 (36.36) | 12 (40) |
| Dysthymia | 4 (13.79) | 3 (13.64) | 3 (10) |
| GAD | 2 (6.90) | 3 (13.64) | 4 (11.11) |
| Panic disorder | 2 (6.90) | 1 (4.55) | 2 (6.67) |
| Agoraphobia | 2 (6.90) | 1 (4.55) | 2 (6.67) |

**Figure S4.** CONSORT diagram of participants' progression through the study.

**
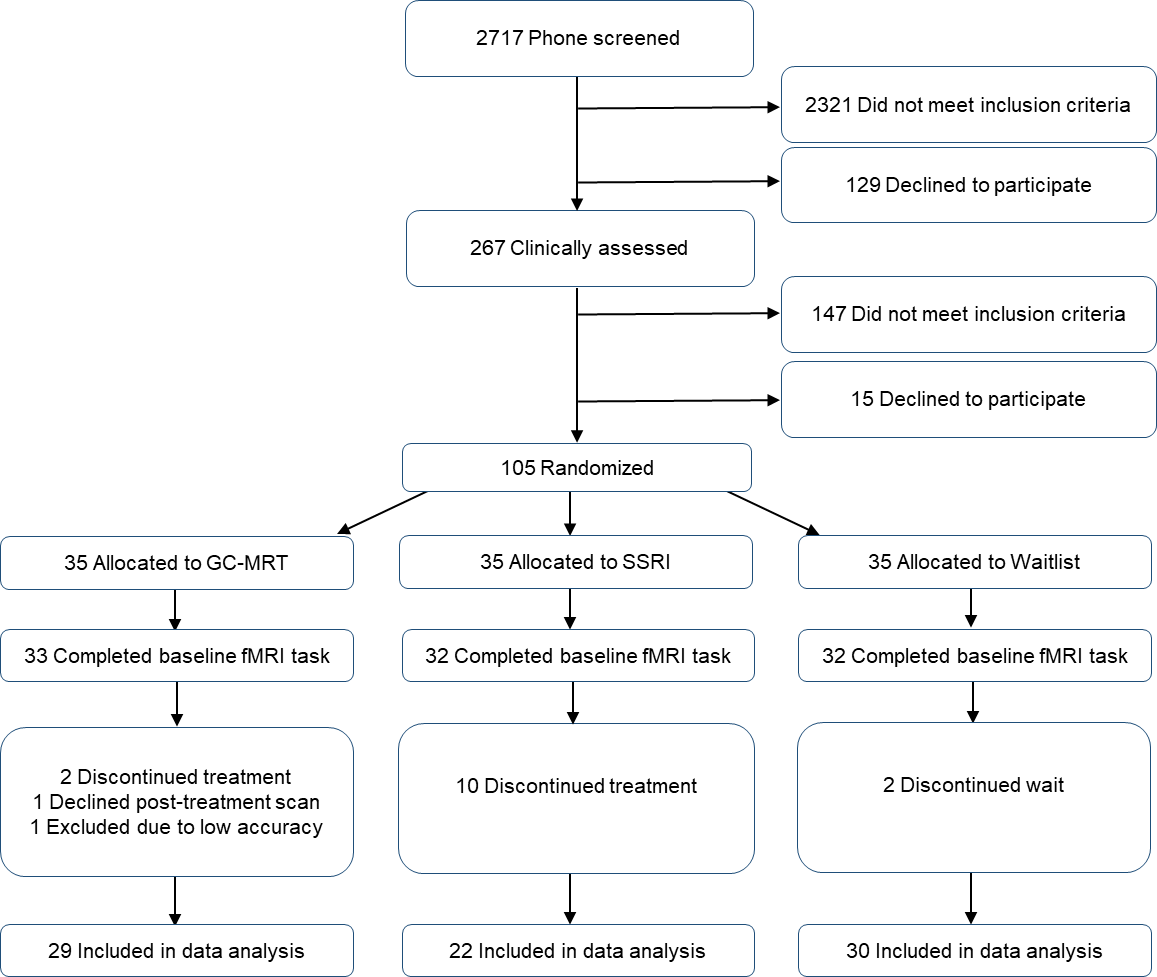
**

**Table S4.** Correlations between symptom change and activation change within brain regions where significant clusters were found in hypothesis 1 and 2 testing. Correlations are presented separately for GC-MRT (A) and SSRI (B) patients. All correlations are not significant (*p*s>.17).

**A**

| **Contrast** | **Analysis** | | **Region** | **Symptom change – activation change Pearson correlation** |
| --- | --- | --- | --- | --- |
| Incongruent to training > Congruent to training | Between-group comparisons | Treatment vs. WL | ACC_2_ | .11 |
|  |  |  | rIFG | .26 |
|  |  | GC-MRT vs. SSRI | ACC_3_ | .09 |

**B**

| **Contrast** | **Analysis** | | **Region** | **Symptom change – activation change Pearson correlation** |
| --- | --- | --- | --- | --- |
| Incongruent to training > Congruent to training | Between-group comparisons | Treatment vs. WL | ACC_2_ | .17 |
|  |  |  | rIFG | .05 |
|  |  | GC-MRT vs. SSRI | ACC_3_ | .13 |

**Figure S5.** Mean BOLD signal change during indirect threat processing (away from threat > away from neutral), within a brain region in which pre-to-post activation increase during direct threat processing (toward threat > toward neutral) was different between treatment (GC-MRT and SSRI) and WL patients. Mean values are presented separately for treated and WL patients (fully colored bars), and GC-MRT and SSRI patients (striped bars). Within groups, all pre-to-post pairwise comparisons indicated no significant effect of time (*p*s>.57).


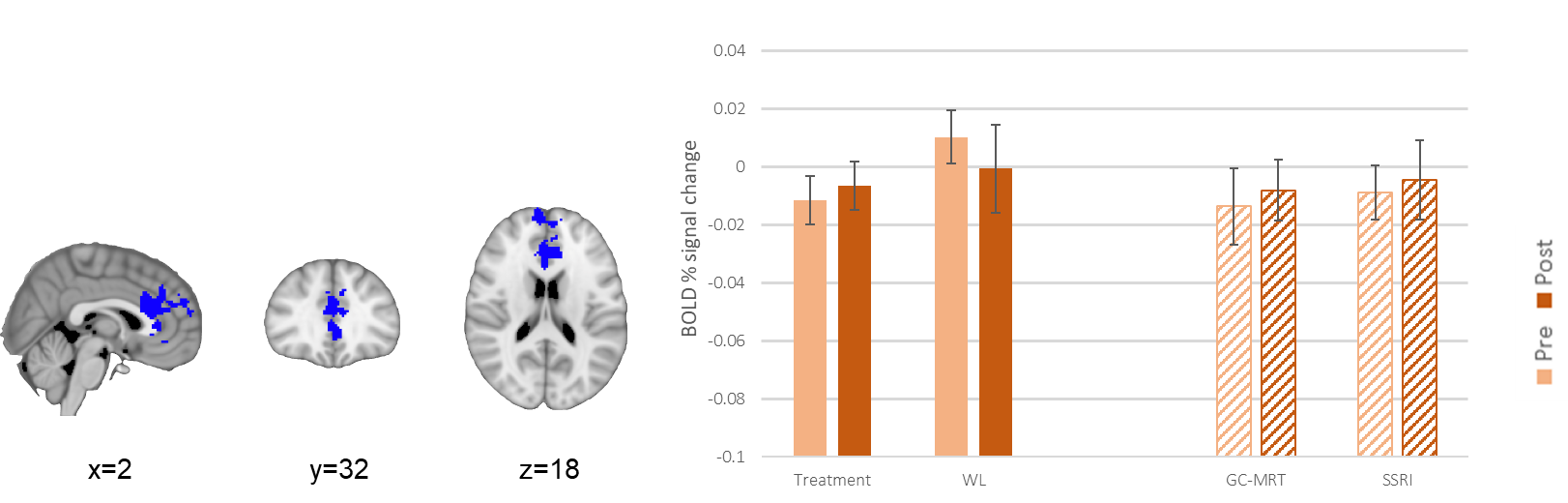


**Clusters Localization**

Locus identification of significant clusters was based on peak coordinates, using reports drawn from the Neurosynth meta-analytic database (http://www.neurosynth.org).

ACC_2_ (Demblon, Bahri, & D’Argembeau, 2016; Kano et al., 2014; Morgan et al., 2015)

rIFG (Friedman et al., 2017; Smyser et al., 2016)

ACC_3_ (Bunford, Roberts, Kennedy, & Klumpp, 2017; Monk et al., 2004)

dlPFC (Goble et al., 2011; Meade, Lowen, MacLean, Key, & Lukas, 2011; Rocca et al., 2014; Wang et al., 2010)

ACC_1_ (Ansado et al., 2013; Waring & Kensinger, 2011)

**References**

Ansado, J., Monchi, O., Ennabil, N., Deslauriers, J., Jubault, T., Faure, S., & Joanette, Y. (2013). Coping with task demand in aging using neural compensation and neural reserve triggers primarily intra-hemispheric-based neurofunctional reorganization. *Neuroscience Research*, *75*(4), 295–304. https://doi.org/10.1016/j.neures.2013.01.012

Beaton, E. A., Schmidt, L. A., Schulkin, J., Antony, M. M., Swinson, R. P., & Hall, G. B. (2009). Different fusiform activity to stranger and personally familiar faces in shy and social adults. *SOCIAL NEUROSCIENCE*, *4*(4), 308–316. https://doi.org/10.1080/17470910902801021

Bunford, N., Roberts, J., Kennedy, A. E., & Klumpp, H. (2017). Neurofunctional correlates of behavioral inhibition system sensitivity during attentional control are modulated by perceptual load. *Biological Psychology*, *127*, 10–17. https://doi.org/10.1016/J.BIOPSYCHO.2017.04.015

Demblon, J., Bahri, M. A., & D’Argembeau, A. (2016). Neural correlates of event clusters in past and future thoughts: How the brain integrates specific episodes with autobiographical knowledge. *NeuroImage*, *127*, 257–266. https://doi.org/10.1016/J.NEUROIMAGE.2015.11.062

Friedman, A. L., Burgess, A., Ramaseshan, K., Easter, P., Khatib, D., Chowdury, A., … Diwadkar, V. A. (2017). Brain network dysfunction in youth with obsessive-compulsive disorder induced by simple uni-manual behavior: The role of the dorsal anterior cingulate cortex. *Psychiatry Research: Neuroimaging*, *260*, 6–15. https://doi.org/10.1016/J.PSCYCHRESNS.2016.12.005

Goble, D. J., Coxon, J. P., van Impe, A., Geurts, M., Doumas, M., Wenderoth, N., & Swinnen, S. P. (2011). Brain Activity during Ankle Proprioceptive Stimulation Predicts Balance Performance in Young and Older Adults. *The Journal of Neuroscience*, *31*(45), 16344. https://doi.org/10.1523/JNEUROSCI.4159-11.2011

Goldin, P. R., Manber, T., Hakimi, S., Canli, T., & Gross, J. J. (2009). Neural bases of social anxiety disorder: Emotional reactivity and cognitive regulation during social and physical threat. *Archives of General Psychiatry*, *66*(2), 170–180. https://doi.org/10.1001/archgenpsychiatry.2008.525

Jenkinson, M., Beckmann, C. F., Behrens, T. E. J., Woolrich, M. W., & Smith, S. M. (2012). FSL. *NeuroImage*, *62*(2), 782–790. https://doi.org/10.1016/J.NEUROIMAGE.2011.09.015

Kano, M., Coen, S. J., Farmer, A. D., Aziz, Q., Williams, S. C. R., Alsop, D. C., … O’Gorman, R. L. (2014). Physiological and psychological individual differences influence resting brain function measured by ASL perfusion. *Brain Structure and Function*, *219*(5), 1673–1684. https://doi.org/10.1007/S00429-013-0593-8/FIGURES/5

Kanwisher, N., & Yovel, G. (2006). The fusiform face area: a cortical region specialized for the perception of faces. *Philosophical Transactions of the Royal Society B: Biological Sciences*, *361*(1476), 2109–2128. https://doi.org/10.1098/RSTB.2006.1934

Kincade, J. M., Abrams, R. A., Astafiev, S. V, Shulman, G. L., & Corbetta, M. (2005). An event-related functional magnetic resonance imaging study of voluntary and stimulus-driven orienting of attention. *Journal of Neuroscience*, *25*(18), 4593–4604. https://doi.org/10.1523/JNEUROSCI.0236-05.2005

Kret, M. E., Pichon, S., Grèzes, J., & De Gelder, B. (2011). Similarities and differences in perceiving threat from dynamic faces and bodies. An fMRI study. *NeuroImage*, *54*(2), 1755–1762. https://doi.org/10.1016/J.NEUROIMAGE.2010.08.012

Lanssens, A., Pizzamiglio, G., Mantini, D., & Gillebert, C. R. (2020). Role of the dorsal attention network in distracter suppression based on features. *Cognitive Neuroscience*, *11*(1–2), 37–46. https://doi.org/10.1080/17588928.2019.1683525

Marinkovic, K., Trebon, P., Chauvel, P., & Halgren, E. (2000). Localised face processing by the human prefrontal cortex: Face-selective intracerebral potentials and post-lesion deficits. *Cognitive Neuropsychology*, *17*(1–3), 187–199. https://doi.org/10.1080/026432900380562

Meade, C. S., Lowen, S. B., MacLean, R. R., Key, M. D., & Lukas, S. E. (2011). fMRI brain activation during a delay discounting task in HIV-positive adults with and without cocaine dependence. *Psychiatry Research: Neuroimaging*, *192*(3), 167–175. https://doi.org/10.1016/J.PSCYCHRESNS.2010.12.011

Monk, C. S., Nelson, E. E., Woldehawariat, G., Montgomery, L. A., Zarahn, E., McClure, E. B., … Pine, D. S. (2004). Experience-dependent plasticity for attention to threat: Behavioral and neurophysiological evidence in humans. *Biological Psychiatry*, *56*(8), 607–610. https://doi.org/10.1016/J.BIOPSYCH.2004.07.012

Monroe, J. F., Griffin, M., Pinkham, A., Loughead, J., Gur, R. C., Roberts, T. P. L., & Christopher Edgar, J. (2013). The fusiform response to faces: Explicit versus implicit processing of emotion. *Human Brain Mapping*, *34*(1), 1–11. https://doi.org/10.1002/HBM.21406

Morgan, J. K., Ambrosia, M., Forbes, E. E., Cyranowski, J. M., Amole, M. C., Silk, J. S., … Swartz, H. A. (2015). Maternal response to child affect: Role of maternal depression and relationship quality. *Journal of Affective Disorders*, *187*, 106–113. https://doi.org/10.1016/J.JAD.2015.07.043

Rocca, M. A., Valsasina, P., Fazio, R., Previtali, S. C., Messina, R., Falini, A., … Filippi, M. (2014). Brain connectivity abnormalities extend beyond the sensorimotor network in peripheral neuropathy. *Human Brain Mapping*, *35*(2), 513. https://doi.org/10.1002/HBM.22198

Smyser, C. D., Snyder, A. Z., Shimony, J. S., Mitra, A., Inder, T. E., & Neil, J. J. (2016). Resting-State Network Complexity and Magnitude Are Reduced in Prematurely Born Infants. *Cerebral Cortex (New York, NY)*, *26*(1), 322. https://doi.org/10.1093/CERCOR/BHU251

Staugaard, S. R. (2010). Threatening faces and social anxiety: A literature review. *Clinical Psychology Review*. https://doi.org/10.1016/j.cpr.2010.05.001

Wang, L., Liu, X., Guise, K. G., Knight, R. T., Ghajar, J., & Fan, J. (2010). Effective Connectivity of the Fronto-parietal Network during Attentional Control. *Journal of Cognitive Neuroscience*, *22*(3), 543–553. https://doi.org/10.1162/JOCN.2009.21210

Waring, J. D., & Kensinger, E. A. (2011). How emotion leads to selective memory: Neuroimaging evidence. *Neuropsychologia*, *49*(7), 1831–1842. https://doi.org/10.1016/j.neuropsychologia.2011.03.007

Yeo, B., Krienen, F. M., Sepulcre, J., Sabuncu, M. R., Lashkari, D., Hollinshead, M., … Buckner, R. L. (2011). The organization of the human cerebral cortex estimated by intrinsic functional connectivity. *Journal of Neurophysiology*, *106*(3), 1125–1165. https://doi.org/10.1152/jn.00338.2011
